# Oscillators and Syllables: A Cautionary Note

**DOI:** 10.3389/fpsyg.2012.00364

**Published:** 2012-10-05

**Authors:** Fred Cummins

**Affiliations:** ^1^UCD School of Computer Science and Informatics, University College DublinDublin, Ireland

There is a belief, widespread in some quarters, that the syllable is usefully thought of as a cyclic or oscillatory system. An oscillatory system allows the definition of phase (or “time” from the point of view of the system) and this in turn allows speculative accounts of entrainment between the bio-mechanical speech production of the speaker and the neurodynamics of the listener. A very explicit account of this nature is provided by Peelle and Davis (2012), and it in turn relies heavily upon a body of work by Poeppel and others, e.g., Luo and Poeppel (2007). These two articles may be taken as representative of a larger literature that shares a common approach to the syllable considered as an oscillatory system. A recent summary is provided in Giraud and Poeppel (2012). In this short note, a cautionary flag will be raised about such accounts from a phonetician's point of view.

The syllable is a construct that is central to our understanding of speech. Very young speakers can be taught to introspect about the syllabic count of their utterances in many instances (Liberman et al., 1974). Some languages base their orthographic systems on the syllable. Musicians associate syllables with discrete notes (Patel and Daniele, 2003). Articulatory movements are made more readily interpretable if we posit the syllable as an organizing (or emergent) structure governing relative timing among discrete effectors (Browman and Goldstein, 1988). The apparent facility with which the syllable is employed in many accounts belies an important observation: syllables are not readily observable in the speech signal. Like their phonological cousins, the phonemes, they make a lot of sense to us as speakers and listeners, but it is not a simple matter to map from this intuition onto either the acoustic signal or the articulatory trace. Even competent adult English speakers may have difficulty counting syllables in a given utterance, and there may be no objective grounds upon which once can answer the difficult question of how many syllables are found in a specific production of an utterance. Just a few illustrative examples encountered by the author recently included such words and phrases as “zoologist” (found to be produced as 2, 3, or 4 syllables), “Carol” (1 or 2), “naturally” (2 and 3), “by his” (1 and 2), etc. Note: ambiguity obtains in attempting to identify the number of syllables in actually produced tokens, not in idealized, imagined ones. Such examples abound once one directs ones attention to specific utterances as spoken. They are not exceptions to a largely unproblematic majority.

Poeppel, Giraud, Peelle, and others lean heavily on the “amplitude envelope” of the speech signal as a supposed carrier of information about syllabic phase, and the modulation of this envelope is supposed to arise from quasi-cyclic wagging of the jaw. Typical syllable rates are observed to lie approximately within the same range as theta oscillations, conventionally delimited to 4–8 Hz. And so the inference arises that modulation of the amplitude envelope, arising in quasi-cyclic jaw wagging, contains syllabic information at a temporal rate matched to known theta oscillations, and, of course, theta oscillation is found to be modulated in part by the amplitude envelope.

**Figure 1:**
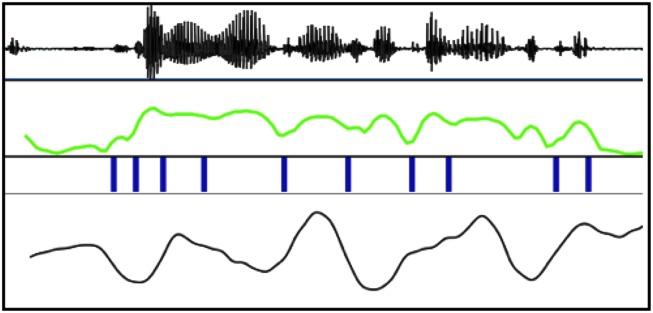
**From top: sound wave, amplitude envelope, approximate syllable boundaries, and first principal component of jaw movement in the mid-saggital plane for one fairly rapid utterance of the Slovak sentence that might be represented canonically thus**: 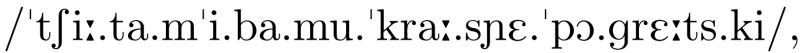 **but in this instance is spoken rather as** 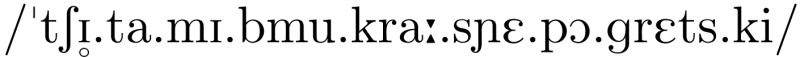

But the wagging of the jaw is not a guide to the unfolding of syllables in sequence, and, even if it were, it is not typically possible to recover jaw position from the amplitude envelope (Beňuš and Pouplier, 2011). The amplitude envelope bears a fiercely complex relationship to the movement of all the articulators, not just the jaw (see Figure 1). It is substantially modulated by all kinds of tongue movement, by lip aperture, and by velar opening and closing. It certainly does not provide unambiguous or even nearly unambiguous information about syllables. Intuitions about syllabic regularity are thus potentially misleading, and a theoretical account that depends upon information about syllabic sequence being present in the amplitude envelope must at the very least demonstrate that that information is, in fact, present. It is certainly not sufficient to point out that a mean syllable rate of about 5 Hz seems to match the entirely conventional range of theta oscillation, nor to observe that although speech is not strictly periodic, it is at least quasi-periodic. Such untempered laxness, it seems to this phonetician, will not serve.

Likewise, the observation that appropriate amplitude envelope modulation is a critical contributor to the intelligibility of speech is neither necessary nor sufficient to shore up a claim that the amplitude envelope provides information about syllables, or that it can serve as the basis for entrainment between speakers and listeners (Ghitza, 2012).

Oscillation in the brain is uncontroversially present at a range of frequencies (Buzsáki and Draguhn, 2004), and furthermore, the temporal modulation of the amplitude envelope of the speech wave, or of a band pass filtered component thereof, may be causally linked to modulation of theta oscillation (Luo and Poeppel, 2007). None of this need be questioned to argue that it is the speech signal itself that is being mischaracterized in such accounts. The speech signal is not periodic in the sense required to support entrainment with the source of theta or gamma oscillations. Furthermore, caution is especially warranted as the term “rhythm” is used in fundamentally different ways within neuroscience – where it is treated as synonymous with “periodic” – and in our every day talk of speech – where rhythm is more akin to musical rhythm, and much harder to define in an objective sense.

An entrainment account based on the amplitude envelope (or the jaw) as the mediating signal that yokes two systems together is fundamentally incomplete. It is incomplete, not because speakers and listeners do not entrain – they do, and there is increasing evidence for coupling at every level – but because such an account omits the *knowledge* that speakers/listeners bring to bear on the exchange (Cummins, 2012). This is perhaps best illustrated by the ability of speakers to speak in extremely close synchrony with one another (Cummins, 2009). In the absence of any periodic grid to support mutual timing registration, speakers can, without effort, align their spoken utterances of a novel text. This is possible, not because the syllables are recoverable from the amplitude envelope. Indeed, it was found that the amplitude envelope was neither necessary nor sufficient to facilitate synchronization among speakers (Cummins, 2009), and that synchronization depended upon a complex suite of interacting factors, among which intelligibility seemed to be the single most important (although intelligibility is not related to any single signal property). On the contrary, close synchronous speaking is possible because speakers *share the knowledge* of the severe spatio-temporal constraints that collectively define what it is to speak a specific language. The coupling exhibited here is, of course, between the neuro-bio-mechanics of one skilled speaker and the neuro-bio-mechanics of the other – like coupling with like. The coupling critically involves the whole of the two speakers, including their skill sets.

There seems to be a need here for the development of formal models that can capture the reciprocal coupling of speaker and listener, taking into account their implicit but hugely constraining practical knowledge of what it is to speak. A mechanical model that treats syllable-producers as oscillators and syllable-hearers as entraining to those oscillations, seems, to this phonetician, to ignore much of the known complexity of speech as she is spoken and of speakers as they speak.
